# Alogliptin Delays the Healing of Traumatic Oral Ulcers in the Buccal Mucosa of Wistar Rats

**DOI:** 10.1111/fcp.70090

**Published:** 2026-04-23

**Authors:** Maria Imaculada de Queiroz Rodrigues, Joyce Ohana de Lima Martins, Debora de Souza Collares Maia Castelo‐Branco, Paulo Goberlânio de Barros Silva, Fabrício Bitu Sousa, Mário Rogério Lima Mota, Ana Paula Negreiros Nunes Alves

**Affiliations:** ^1^ Department of Dental Clinic, Stomatology and Oral Pathology Sector, School of Pharmacy, Dentistry and Nursing Federal University of Ceara Fortaleza Ceará Brazil; ^2^ Applied Group in Medical Microbiology, Postgraduate Program in Medical Microbiology, Faculty of Medicine Federal University of Ceara Fortaleza Ceará Brazil; ^3^ Department of Dentistry Unichristus Fortaleza Ceará Brazil

**Keywords:** dipeptidyl peptidase IV inhibitors, oral ulcers, toll‐like receptor 4, wound healing

## Abstract

**Objective:**

The objective of this study is to evaluate the influence of alogliptin treatment on the healing process of traumatic oral ulcers.

**Methods:**

Four experimental groups were used: control group (GC) and three test groups treated with oral Alogliptin at 1 (GTA1), 3 (GTA3), and 9 mg/kg/day (GTA9). Ulcer diameter, body weight, glycemic index, colony‐forming unit (CFU), and discomfort were analyzed. Histological slides were prepared for healing scores, inflammatory cell counts, collagen deposition analysis, and immunohistochemistry.

**Results:**

Alogliptin treatment increased ulcer area (GTA3‐7D: 5.2 ± 1.2; GTA9‐3D: 11.8 ± 0.8; GTA9‐7D: 5.8 ± 1.3; *p* < 0.001), CFU counts (*p* = 0.049), and Grimace/discomfort scores (*p* = 0.02), while reducing body weight gain (*p* = 0.007) in GTA3 and GTA9 groups. Microscopic analysis revealed higher histopathological scores (*p* = 0.039), increased mononuclear cells (*p* = 0.006), reduced polymorphonuclear cells (*p* < 0.05), and decreased collagen deposition (18.2 ± 2.6; *p* = 0.031) in GTA9. Lower TLR4 (*p* = 0.001) and TGF‐β (*p* < 0.001) expression, alongside increased CD31 immunostaining (*p* < 0.001), were observed in GTA3 and GTA9, as well as reduced TLR2 expression (*p* = 0.001) in GTA9.

**Conclusion:**

Alogliptin delays oral ulcer healing by sustaining inflammation, reducing TGF‐β expression, and impairing collagen deposition, and may contribute via reduced TLR2/TLR4 expression, increased microbial burden, and decreased TGF‐β.

## 1 Introduction

Oral ulcers are a common condition, frequently reported by patients, and cause pain and discomfort. These lesions are characterized by epithelial discontinuity, covered by a fibrinopurulent membrane with an erythematous border. The primary cause of such ulcers is trauma, hence termed traumatic ulcers [1, 2].

Wound healing, as seen in oral ulcers, is a complex process that occurs in all human tissues. However, the proper progression of healing phases can be disrupted by factors such as microbial infection, which may secondarily colonize ulcers. Evidence indicates that microorganisms colonize wound surfaces and form communities known as biofilms [3]. Following tissue injury, exposure to the oral environment renders the wound susceptible to colonization by the diverse oral microbiota. An insufficient host response allows these microorganisms to invade the wound site, proliferate, and establish infection. This process intensifies and extends inflammation by promoting excessive production of pro‐inflammatory cytokines, including interleukin (IL)‐1 and tumor necrosis factor (TNF)‐α, which ultimately delays healing [3, 4, 5].

The inflammatory response to microorganisms is mediated by pattern recognition receptors (PRRs), expressed on immune cells such as macrophages, dendritic cells, and neutrophils. TLR4, a key PRR, recognizes bacterial lipopolysaccharide (LPS) from gram‐negative bacteria and endogenous molecules released during inflammatory or infectious disorders [6, 7].

The oral cavity hosts over 750 bacterial species colonizing various niches. Under equilibrium, these microorganisms coexist symbiotically with the host. However, microenvironmental changes can trigger infectious processes [8, 9]. Although the oral microbiota of laboratory animals is predominantly composed of gram‐positive bacteria (90%), a smaller population of potentially pathogenic gram‐negative bacteria, such as *Escherichia coli* , is also present [10]. Gram‐negative bacteria are frequently associated with surgical wound infections due to endotoxin production, which enhances their virulence [11].

Bacterial recognition is critical for initiating the host immune response [6, 7]. Failure in this process increases susceptibility to local pathogens, enabling bacterial colonization, infection, and delayed healing [3, 6].

Alogliptin, a dipeptidyl peptidase‐4 (DPP‐4) inhibitor, is a widely prescribed antidiabetic drug that may also modulate inflammatory pathways relevant to wound healing. Preclinical and clinical studies suggest that alogliptin and other DPP‐4 inhibitors indirectly suppress TLR4‐mediated signaling and downregulate TLR4 expression, though this is not their primary mechanism of action [12, 13, 14, 15, 16].

Thus, impaired bacterial recognition via TLR4 downregulation may increase susceptibility to infections in wounds such as oral ulcers. This could amplify inflammatory responses through alternative pathways, delaying healing. While microbial recognition has been investigated in cutaneous wound healing, its role in traumatic oral ulcer healing remains unexplored. Therefore, this study aimed to evaluate the influence of alogliptin on the healing of traumatic oral ulcers in the buccal mucosa of Wistar rats.

## 2 Materials and Methods

### 2.1 Ethical Aspects

This study was approved by the Animal Ethics Committee (CEUA) of the Federal University of Ceará (Protocol No. 9982290120). The work adhered to the ARRIVE guidelines (Animal Research: Reporting In Vivo Experiments) [17] and ethical practices to minimize animal discomfort, following the 3R principles (reduction, refinement, and replacement).

### 2.2 Animals and Sample Size Calculation

The study was conducted at the Oral Pathology Laboratory (LPB) of the Graduate Program in Dentistry (PPGO) at the Federal University of Ceará (UFC). Female Wistar rats (180–220 g) were housed in the experimental vivarium of the Department of Physiology and Pharmacology (DFF‐UFC) under controlled conditions (24°C, 12‐h light–dark cycle) with ad libitum access to water and food. Based on a previous insulin resistance model [18], which reported significant behavioral improvement in rats treated with alogliptin (11 ± 7 to 25 ± 10), a sample size of six animals per group was determined to achieve 80% power and 95% confidence.

### 2.3 Experimental Groups and Ulcer Induction Protocol

Four groups were established: GC: 0.1‐mL/kg/day saline solution; GTA1, GTA3, andGTA9: alogliptin at 1, 3, and 9 mg/kg/day, respectively, dissolved in saline [19]. Doses of 1 and 3 mg/kg/day inhibit DPP‐4 in rats, equivalent to a human dose of 25 mg/day for a 60‐kg adult [20]. The supra‐therapeutic dose of 9 mg/kg/day was added for dose–response analysis. Treatment began 3 days pre‐ulcer induction and continued until euthanasia.

Rats were randomly assigned to groups using Microsoft Excel's RAND() function by a researcher other than the operator, anesthetized intraperitoneally with 10% ketamine (80 mg/kg) and 2% xylazine (10 mg/kg), and subjected to mucosal antisepsis (0.12% chlorhexidine). A 6‐mm diameter, 2‐mm deep ulcer was induced in the left buccal mucosa using a dermatological punch (Rhosse), followed by excision of residual tissue with a Bard‐Parker scalpel [21]. Post‐procedure, animals were wrapped in bubble wrap and monitored in warm cages (using gloves filled with warm water) to prevent hypothermia. No dietary or hydration restrictions were imposed.

Animals were euthanized on Days 1, 3, 7, and 14 post‐ulcer induction (*n* = 6/group/day), totaling 96 animals (Table 1).

**TABLE 1:** Distribution of animals by experimental groups and days of euthanasia.

	Day 1	Day 3	Day 7	Day 14	Total
Saline	*n* = 6	*n* = 6	*n* = 6	*n* = 6	*n* = 24
1 mg/kg	*n* = 6	*n* = 6	*n* = 6	*n* = 6	*n* = 24
3 mg/kg	*n* = 6	*n* = 6	*n* = 6	*n* = 6	*n* = 24
9 mg/kg	*n* = 6	*n* = 6	*n* = 6	*n* = 6	*n* = 24
**Total**	*n* = 24	*n* = 24	*n* = 24	*n* = 24	*n* = 96

### 2.4 Discomfort Analysis

Animals euthanized on Day 14 were assessed daily for discomfort using the Grimace Scale. Rats were individually placed in a dark room with red light in polypropylene boxes. After 5 min of acclimatization, they were observed for 5 min, scoring orbital tightening, nose/cheek bulging, ear position, and whisker changes (0: no discomfort; 1: mild; 2: severe). Results were expressed as median (min–max). Area under the curve (AUC) was calculated for each animal and compared between groups [22]. The use of analgesics was not considered, as it could be a confounding factor. However, severe changes in animal behavior would be a parameter for establishing a humane endpoint through euthanasia due to anesthetic overdose.

### 2.5 Measurement of Lesion Diameter, Body Weight Variation, and Glycemic Index

Euthanasia was performed via anesthetic overdose (3× ketamine: 240 mg/kg; xylazine: 30 mg/kg). Post‐euthanasia, ulcer swabs were collected for microbiological analysis. Ulcer area was measured using a digital caliper (Digimess; precision: 0.01 mm), with two diameters (D: major; d: minor) used to calculate area (A = π·d·D/4). Body weight variation (Δweight = final − initial) and blood glucose (50 μL collected from the caudal vein, Accu Chek glucometer) were recorded [23].

### 2.6 Microbiological Analysis of Ulcer Surface

Ulcer surfaces were swabbed with sterile microbrushes before treatment initiation and at each euthanasia time point. Swabs were placed in 1 mL of 0.89% buffered saline [24], vortexed (AP56, Phoenix Luferco) for 1 min, and serially diluted (1:10, 1:100, 1:1000). Aliquots (25 μL) were plated on Brain Heart Infusion (BHI) agar (Kasvi) and incubated at 37°C for 48 h. Colony‐forming units (CFU/mL) were counted.

### 2.7 Histological Analysis

Ulcerated mucosal tissue was excised, fixed in 10% buffered formalin for 48 h, and processed for histology. Twenty‐four stained slides (hematoxylin‐eosin) were scored microscopically: 0: No ulcer, remodeled connective tissue; 1: No ulcer, mild/moderate fibrosis with chronic inflammation; 2: Ulcer with fibrosis and moderate chronic inflammation; 3: Ulcer with chronic inflammation (granulation tissue); 4: Ulcer with acute inflammation (ectasia, dilated vessels, and mixed infiltrate) [1].

### 2.8 Histomorphometric Analysis: Evaluation of Inflammatory Infiltrate

Five microscopic fields near the ulcerated region (three superficial, two deep; total area: 1.30 mm^2^) were photographed from hematoxylin‐eosin‐stained slides at 400× magnification using a Leica DM2000 microscope equipped with a DFC295 camera and LAS software. Total counts of polymorphonuclear neutrophils (PMNs) and mononuclear cells were performed using ImageJ's Cell Counter tool, with the sum used as the sampling unit. Results were expressed as mean ± standard error of the mean (SEM) [23].

### 2.9 Histochemical Analysis: Collagen Deposition

For total collagen analysis, 3‐μm sections were stained with Masson's Trichrome and Picrosirius Red.

Masson's Trichrome: Five fields per slide were photographed and analyzed in ImageJ using color deconvolution (Color function > Color deconvolution > Alcian blue & H) to isolate blue (collagen), red, and residual color channels. Blue images were binarized (Process > Binary > Make Binary), and the percentage of collagen‐positive area was calculated (Analyze > Analyze Particles) [25].

Picrosirius Red: To quantify red‐stained collagen, images were thresholded (Image > Adjust > Color Threshold) with RGB parameters (red: 71–255; green: 0–69; blue: 0–92). Afterwards, the images were converted to the 8‐bit color scale (Image > Type > 8‐bit) and then binarized (Process > Binary > Make Binary). Finally, the percentage of collagen area marked in red was measured (Analyse > Analyze Particles). Results from both methods were averaged across fields and expressed as mean ± SEM.

### 2.10 Immunohistochemical Analysis Using Tissue Microarray (TMA)

Two representative areas (duplicates) from each tissue block were used to construct TMAs (Quick‐Ray UNITMA device). Sections (3 μm) were mounted on silanized slides, and immunohistochemistry was performed using the streptavidin‐biotin technique with overnight incubation of primary antibodies: anti‐TLR4, anti‐TLR2, anti‐TGF‐β, and anti‐CD31. Table 2 describes the dilution and positive control of each antibody. The negative control was performed by suppressing the primary antibody from the reaction in one of the sections.

**TABLE 2:** Specification of primary antibodies.

Antibody	Brand	Host	Clone	Dilution	Antigen retrieval	Positive control
Anti‐TLR4	Abcam	Mouse	Ab22048	1:150	Citrate pH 6.0	Ulcer
Anti‐TLR2	Abcam	Rabbit	Ab213676	1:150	Citrate pH 6.0	Ulcer
Anti‐TGF‐β	Abcam	Rabbit	Ab92486	1:200	Citrate pH 6.0	Ulcer
Anti‐CD31	Abcam	Rabbit	Ab182981	1:1000	Tris EDTA pH 9.0	Rat kidney

*Source:* Prepared by the authors.

A quantitative assessment was performed by two independent observers. Five fields (anti‐TLR4, anti‐TLR2, and anti‐TGF‐β) or three fields (anti‐CD31) were photographed in connective/granulation tissue near the ulcer. Immunostained cells/vessels were counted using ImageJ's Cell Counter. Results were expressed as mean ± SEM [23, 26].

### 2.11 Statistical Analysis

Quantitative data are presented as mean ± SEM (normality tested via Shapiro–Wilk). Grimace scores and histopathological scores are expressed as median (min–max). Parametric data were analyzed using two‐way ANOVA with Bonferroni post hoc test; nonparametric data and scores were analyzed via Kruskal–Wallis/Dunn's test. All analyses were performed in GraphPad Prism 5.0 (95% confidence interval).

## 3 Results

### 3.1 Effect of Alogliptin on Clinical and Microbiological Parameters of Oral Ulcers and Body Weight Variation

Regarding ulcer area, no differences were observed between groups on Day 1. On Day 3, the ulcer area in the GTA9 group (11.8 ± 0.8 mm^2^) was significantly larger than in the control group (6.4 ± 1.0 mm^2^). Similarly, on Day 7 post‐ulceration, the GTA3 (5.2 ± 1.2 mm^2^) and GTA9 (5.8 ± 1.3 mm^2^) groups also showed larger ulcer areas compared to the saline group (1.3 ± 0.6 mm^2^) (*p* < 0.001) (Table 3).

**TABLE 3:** Clinical healing profile of the ulcer, body weight variation, and microbiological count of the ulcer surface in the buccal mucosa of Wistar rats treated with alogliptin.

	Saline	Alogliptin dosage			*p*‐value
		1 mg/kg	3 mg/kg	9 mg/kg	
**Ulcer area (mm** ^**2**^ **)**					
1D	9.4 ± 0.5	12.0 ± 2.3	12.1 ± 1.1	12.7 ± 0.9	**< 0.001** ^**a**^
3D	6.4 ± 1.0	6.5 ± 1.4	6.2 ± 0.8	**11.8 ± 0.8** *	
7D	1.3 ± 0.6	2.6 ± 1.0	**5.2 ± 1.2** *	**5.8 ± 1.3** *	
14D	1.1 ± 0.5	0.8 ± 0.4	1.1 ± 0.4	1.1 ± 0.6	
**Body weight (g)**					
1D	97.8 ± 0.9	98.7 ± 1.3	97.6 ± 0.6	96.7 ± 1.0	**0.007** ^a^
3D	97.0 ± 0.5	96.9 ± 0.3	98.1 ± 0.9	98.1 ± 0.9	
7D	101.6 ± 1.0	98.2 ± 0.9	100.0 ± 1.6	98.9 ± 1.2	
14D	104.7 ± 2.0	103.7 ± 2.0	**98.9 ± 0.8** *	**96.2 ± 1.3** *	
**Microbiological analysis (log**_**10**_ **UFC/mL)**					
D‐3	1.6 ± 0.1	2.2 ± 0.3	1.6 ± 0.5	1.1 ± 0.4	**0.049** ^a^
1D	1.2 ± 0.3	2.2 ± 0.3	2.2 ± 0.4	**2.7 ± 0.2** *	
3D	2.0 ± 0.4	2.6 ± 0.5	**3.6 ± 0.3** *	**3.8 ± 0.5** *	
7D	2.1 ± 0.3	1.8 ± 0.3	2.4 ± 0.6	2.1 ± 0.6	
14D	1.3 ± 0.4	1.9 ± 0.4	1.3 ± 0.3	1.4 ± 0.5	

*Source:* Research data from the study.

^a^ Two‐way ANOVA/Bonferroni test.

* *p* < 0.05 versus saline.

Body weight gain was significantly lower in the GTA3 (98.9 ± 0.8 g) and GTA9 (96.2 ± 1.3 g) groups compared to the saline group (104.7 ± 2.0 g) on Day 14 (*p* = 0.007) (Table 3).

In microbiological analysis, no differences were observed between groups 3 days before ulcer induction. On Day 1 post‐ulceration, the GTA9 group (2.7log_10_ CFU/mL ± 0.2) had significantly higher bacterial counts than the control group (1.2 log_10_ CFU/mL ± 0.3). By Day 3, the GTA3 (3.6 log_10_ CFU/mL ± 0.3) and GTA9 (3.8 log_10_ CFU/mL ± 0.5) groups also showed significantly higher counts compared to the saline group (2.0 log_10_ CFU/mL ± 0.4) (*p* = 0.049) (Table 3).

No statistically significant differences in glycemia were observed between groups throughout the experimental protocol (*p* = 0.075).

### 3.2 Discomfort Analysis

On Days 1, 2, and 5, the median Grimace scores were significantly higher in the GTA9 group compared to the saline group (*p* = 0.014, *p* = 0.01, and *p* = 0.039, respectively). On Day 3, the GTA1 group (*p* = 0.012) showed a significantly lower median score compared to the control group. On Days 10, 12, and 14, the GTA3 group exhibited significantly higher median scores compared to the control group (*p* = 0.005, *p* = 0.025, and *p* = 0.015, respectively) (Table 4).

**TABLE 4:** Discomfort analysis according to the Grimace Scale in Wistar rats treated with alogliptin.

	Saline	Alogliptin dosage			*p‐ value*
		1 mg/kg	3 mg/kg	9 mg/kg	
**Grimace Scale scores**					
**D0**	0 (0–0)	0 (0–0)	0 (0–0)	0 (0–0)	1.000
**D1**	1 (0–2)	2 (1–2)	1.5 (1–2)	**2 (2–4)** *	**0.014** ^**a**^
**D2**	1 (1–2)	1.5 (1–2)	2 (1–2)	**3 (2–3)** *	**0.01** ^**a**^
**D3**	2 (1–2)	**1 (1–1)** *	2 (1–2)	2 (1–3)	**0.012** ^**a**^
**D4**	2 (1–2)	2 (1–3)	1 (1–2)	2 (1–3)	0.135
**D5**	1.5 (1–2)	2 (1–2)	2 (1–2)	**2.5 (2–3)** *	**0.039** ^**a**^
**D6**	1.5 (1–2)	1 (1–2)	1 (1–2)	2 (1–3)	0.13
**D7**	1 (0–2)	1 (1–1)	1 (1–2)	1.5 (0–2)	0.474
**D8**	1 (1–1)	1 (1–2)	1 (1–1)	1 (0–3)	0.764
**D9**	0.5 (0–1)	1 (1–1)	1 (0–2)	1 (0–2)	0.345
**D10**	0 (0–0)	1 (0–1)	**1 (1–2)** *	0 (0–2)	**0.005** ^**a**^
**D11**	0 (0–1)	0.5 (0–1)	1 (0–2)	0 (0–1)	0.285
**D12**	0 (0–0)	0 (0–0)	**1 (0–1)** *	0 (0–1)	**0.025** ^**a**^
**D13**	0 (0–1)	0 (0–0)	0 (0–1)	0 (0–0)	0.554
**D14**	0 (0–0)	0 (0–0)	**1 (0–1)** *	0 (0–1)	**0.015** ^**a**^

*Source:* Research data from the study.

^a^ Kruskal–Wallis/Dunn test.

* *p* < 0.05 versus saline.

**FIGURE 1:**
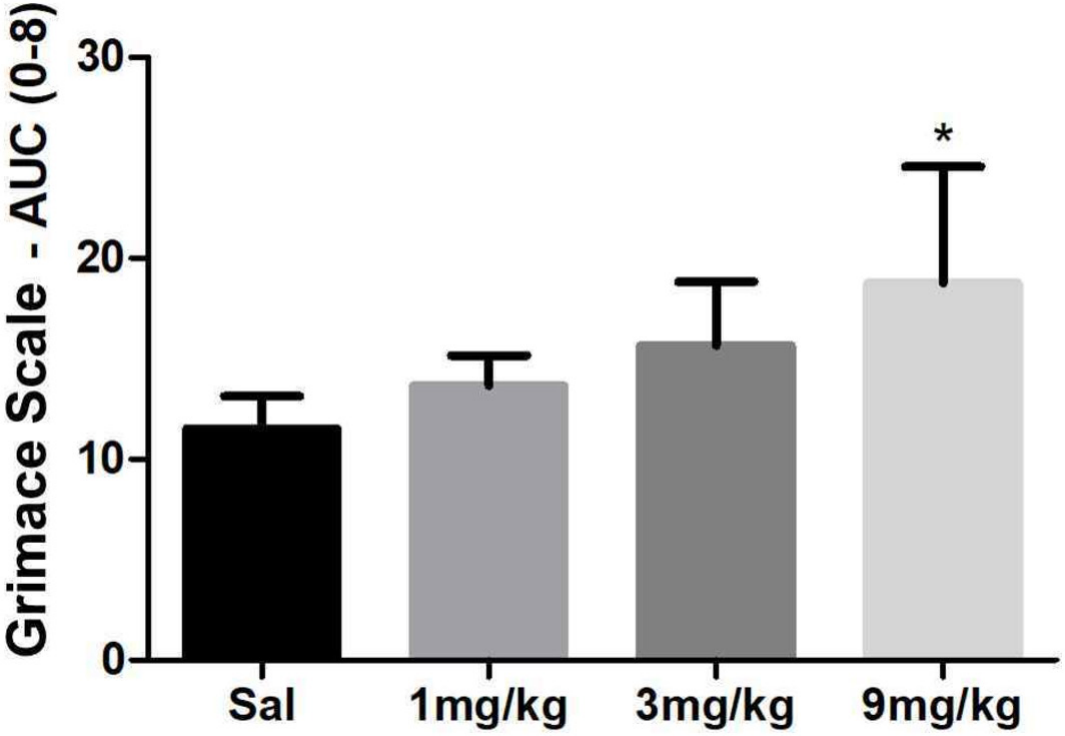
Area under the curve for discomfort analysis according to the Grimace Scale in Wistar rats treated with alogliptin. *Source:* Research data from the study. *p < 0.05 versus saline (mean ± SEM; Kruskal–Wallis/Dunn).

Additionally, when calculating the AUC for each animal, the GTA9 group showed a significantly higher mean AUC for Grimace scores compared to the saline group (*p* = 0.02) (Figure 1).

### 3.3 Effect of Alogliptin on Histopathological Alterations in Oral Ulcers

**FIGURE 2:**
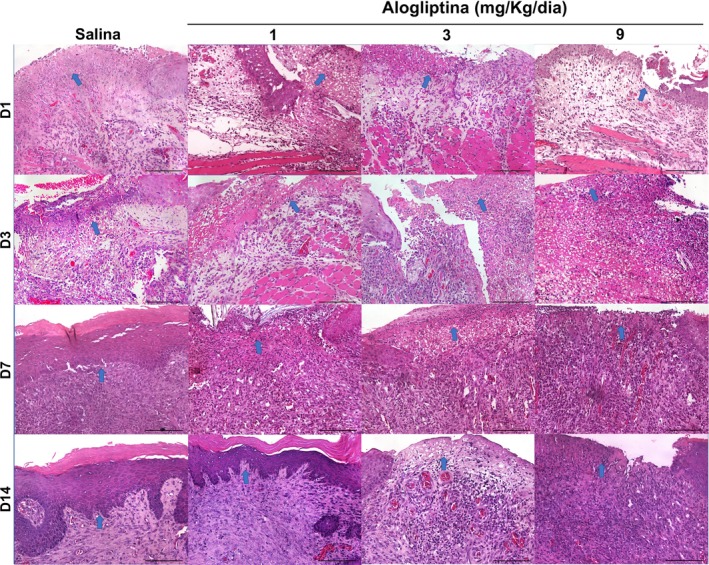
Microscopic profile of ulcers in the buccal mucosa of Wistar rats treated with alogliptin. *Source:* Data from the study. Staining = hematoxylin‐eosin; magnification = 200×; scale bar: 100 μm; blue arrow: Eulcerated/scarred area.

Histological scores significantly decreased over time in all groups. On Day 1, the median score was 4 in all groups, with no differences between groups (*p* = 1.000). On Day 3, scores ranged between 3 and 4, again with no significant differences. On Day 7, the GTA1, GTA3, and GTA9 groups exhibited significantly higher median scores compared to the saline group (*p* < 0.001). By Day 14, the GTA3 and GTA9 groups showed significantly higher median scores compared to the control group (*p* = 0.001) (Figure 2 and Table 5).

**TABLE 5:** Histological scores and histomorphometric analysis in induced ulcer in the buccal mucosa of Wistar rats treated with alogliptin.

	Saline	Alogliptin dosage			*p*‐value
		1 mg/kg	3 mg/kg	9 mg/kg	
**Histological scores**					
1D	4 (4–4)	4 (4–4)	4 (4–4)	4 (4–4)	1.000
3D	4 (3–4)	3 (3–3)	3 (3–4)	4 (3–4)	*0.065*
7D	**1 (1–2)** ^**†**^	**3 (2–3)** *	**3 (2–3)** * ^**†**^	**3 (3–3)** * ^**†**^	**< 0.001** ^**a**^
14D	**0 (0‐1)** ^**†**^	1 (1–2)^†^	**3 (2–3)** * ^**†**^	**3 (2–4)** * ^**†**^	**< 0.001** ^**a**^
***p*‐value** ^**a**^	**< 0.001**	**< 0.001**	**0.001**	**0.004**	
**PMN cells**					
1D	590.8 ± 167.6	617.7 ± 149.1	594.3 ± 71.8	675.3 ± 146.1	**< 0.05** ^**b**^
3D	594.2 ± 117.4	450.2 ± 94.9	340.0 ± 57.0	397.3 ± 44.4	
7D	496.5 ± 68.2	326.8 ± 44.9	221.0 ± 46.6	**139.0 ± 24.8** *	
14D	134.5 ± 51.7	70.4 ± 45.6	99.6 ± 38.8	114.3 ± 42.9	
**Mononuclear cells**					
1D	310.0 ± 38.4	272.7 ± 36.1	242.6 ± 32.8	190.3 ± 20.0	**0.006** ^**b**^
3D	507.7 ± 59.5	595.7 ± 51.1	**708.7 ± 73.8** *	**752.8 ± 37.7** *	
7D	150.8 ± 17.3	196.8 ± 20.8	213.8 ± 32.8	229.2 ± 21.3	
14D	227.8 ± 47.1	163.0 ± 32.6	171.5 ± 42.0	224.2 ± 54.4	

*Source:* Research data from the study.

^a^ Kruskal–Wallis/Dunn test.

^b^ Two‐way ANOVA/Bonferroni test.

* *p* < 0.05 versus saline.

^†^ *p* < 0.05 versus D1.

The control (*p* < 0.001), GTA3 (*p* = 0.001), and GTA9 (*p* = 0.004) groups showed reduced histological scores on Day 7 compared to Day 1. However, in the GTA1 group, this reduction was only observed by Day 14 (*p* < 0.001) (Figure 2 and Table 5).

### 3.4 Histomorphometric Study

The analysis shows that intra‐rater agreement was substantial, with a Cohen's Kappa coefficient of κ = 0.71 (95% CI: 0.65–0.91, *p* < 0.001).

### 3.5 Cellular Profile: PMNs and Mononuclear Cells

PMN counts peaked on Day 1 post‐ulceration and declined over time in all groups. However, on Day 7, the GTA9 group (139.0 ± 24.8 cells/mm^2^) showed a significantly lower count compared to the saline group (496.5 ± 68.2 cells/mm^2^) (*p* < 0.05) (Figure 2 and Table 5).

Mononuclear cell counts peaked on Day 3 in all groups. On the same day, the GTA3 (708.7 ± 73.8 cells/mm^2^) and GTA9 (752.8 ± 37.7 cells/mm^2^) groups exhibited significantly higher counts compared to the saline group (507.7 ± 59.5 cells/mm^2^) (*p* = 0.006) (Figure 2 and Table 5).

### 3.6 Collagen Deposition Analysis

No significant differences in total collagen were observed 7 days post‐ulceration. However, on Day 14, the GTA9 group (18.2% ± 2.6%) showed a significant reduction in collagen deposition compared to the saline group (35.2% ± 0.8%) in Masson's Trichrome analysis (*p* = 0.031) (Table 6). Picrosirius Red staining confirmed this result (*p* = 0.032) (Table 6).

**TABLE 6:** Percentage of collagen deposition area in induced ulcer in the buccal mucosa of Wistar rats treated with alogliptin.

	Saline	Alogliptin dosage			*p*‐value
		1 mg/kg	3 mg/kg	9 mg/kg	
**Percentage of collagen deposition area (Masson's Trichrome)**					
7D	37.3 ± 3.8	32.4 ± 5.1	34.5 ± 4.7	29.4 ± 3.9	**0.031** ^**a**^
14D	35.2 ± 0.8	30.1 ± 4.0	24.4 ± 3.5	**18.2 ± 2.6** *	
**Percentage of collagen deposition area (Picrosirius Red)**					
7D	14.8 ± 3.1	16.0 ± 3.6	12.0 ± 1.2	10.3 ± 2.8	**0.032** ^**a**^
14D	51.8 ± 9.7	36.8 ± 2.6	34.7 ± 3.7	**27.7 ± 4.8** *	

*Source:* Research data from the study.

^a^ Two‐way ANOVA 2/Bonferroni.

* *p* < 0.05 versus saline.

### 3.7 Immunohistochemical Analysis

**FIGURE 3:**
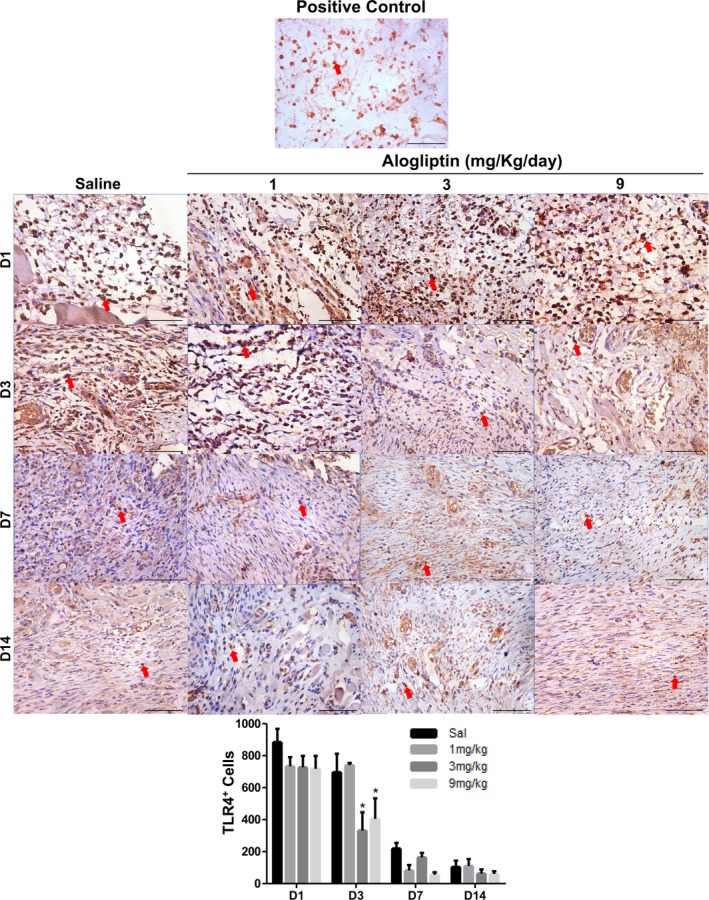
Immunoexpression of TLR4 in ulcerated buccal mucosa of Wistar rats treated with alogliptin. *Source:* Data from the study. Staining = immunohistochemistry; magnification = 400×; scale bar: 50 μm. Red arrow: immunolabeled cell. *p < 0.05 versus saline (mean ± SEM; Two‐way ANOVA/Bonferroni test).

TLR4 expression peaked on Day 1 in all groups except GTA1, where it peaked on Day 3. On Day 3, the GTA3 (332.0 ± 114.2 cells/mm^2^) and GTA9 (404.7 ± 129.4 cells/mm^2^) groups exhibited significantly lower mean TLR4 immunostaining compared to the control group (697.0 ± 115.2 cells/mm^2^) (*p* = 0.001). No differences were observed on Days 7 and 14 (Figure 3 and Table S1).

**FIGURE 4:**
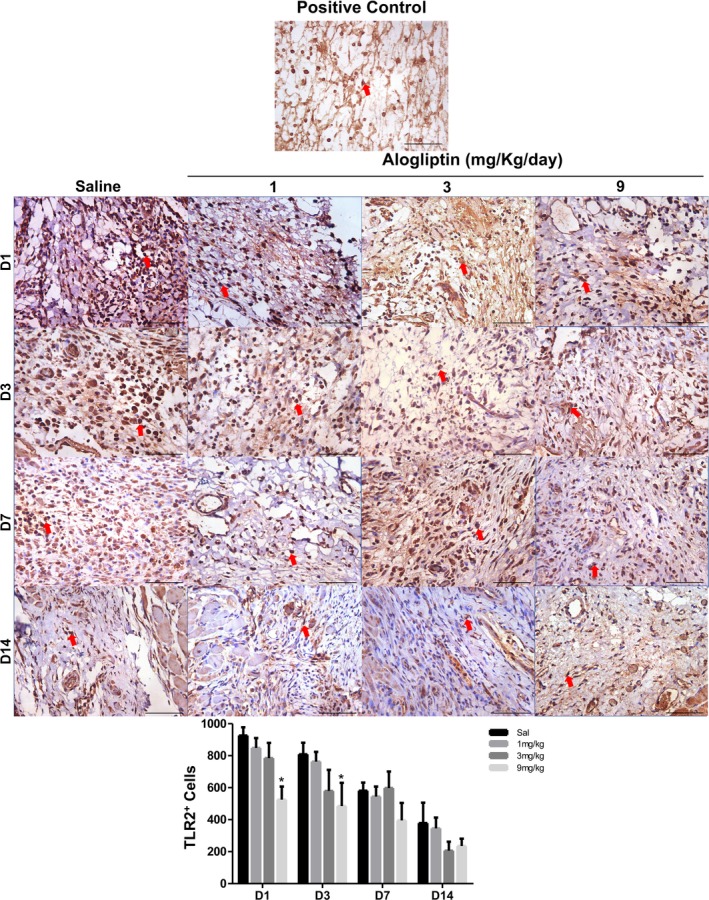
Immunoexpression of TLR2 in ulcerated buccal mucosa of Wistar rats treated with alogliptin. *Source:* Data from the study. Staining = immunohistochemistry; magnification = 400×; scale bar: 50 μm. Red arrow: immunolabeled cell. *p < 0.05 versus saline; (mean ± SEM; Two‐way ANOVA/Bonferroni test).

TLR2 expression: Peak expression occurred on Day 1 in all groups. On Days 1 and 3, the GTA9 group (525.4 ± 82.0 and 485.0 ± 145.6 cells/mm^2^, respectively) showed significantly lower mean TLR2 immunostaining compared to the saline group (926.5 ± 51.0 and 808.5 ± 73.0 cells/mm^2^, respectively) (*p* = 0.001). No differences were observed on Days 7 and 14 (Figure 4 and Table S1).

**FIGURE 5:**
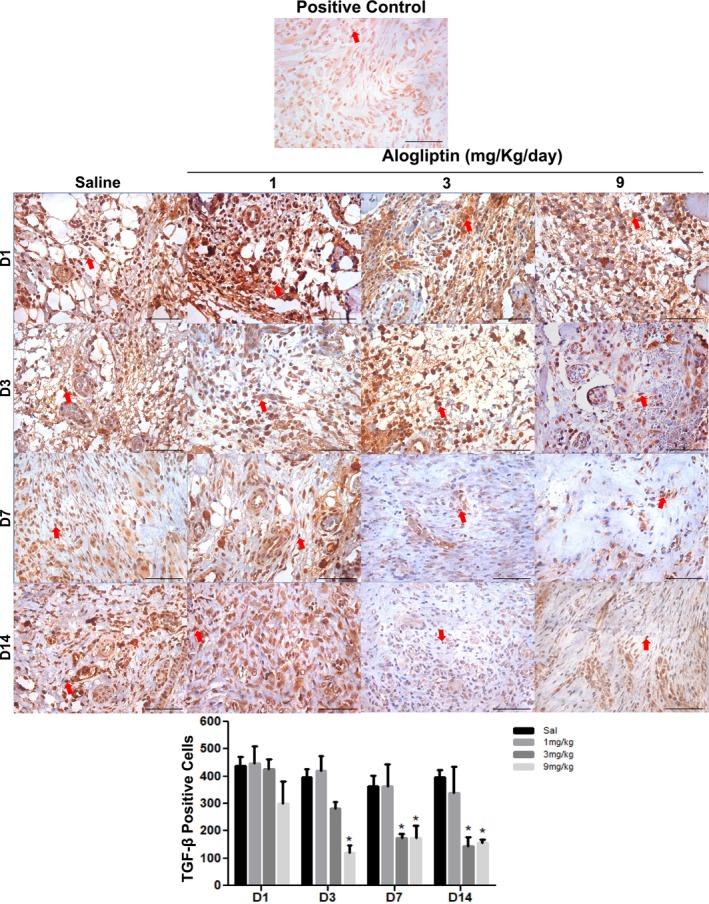
Immunoexpression of TGF‐β in ulcerated buccal mucosa of Wistar rats treated with alogliptin. *Source:* Data from the study. Staining = immunohistochemistry; magnification = 400×; scale bar: 50 μm. Red arrow: immunolabeled cell. *p < 0.05 versus saline; (mean ± SEM; Two‐way ANOVA/Bonferroni test).

TGF‐β expression: Peak expression occurred on Day 1 in all groups. On Day 3, the GTA9 group (121.2 ± 25.3 cells/mm^2^) showed significantly lower TGF‐β immunostaining compared to the saline group (397.3 ± 30.0 cells/mm^2^). By Days 7 and 14, the GTA3 (173.5 ± 16.1 and 145.0 ± 33.4 cells/mm^2^) and GTA9 (175.5 ± 43.6 and 155.0 ± 14.9 cells/mm^2^) groups maintained significantly lower immunostaining compared to the control group (362.4 ± 39.0 and 395.7 ± 26.5 cells/mm^2^, respectively) (*p* < 0.001) (Figure 5 and Table S1).

**FIGURE 6:**
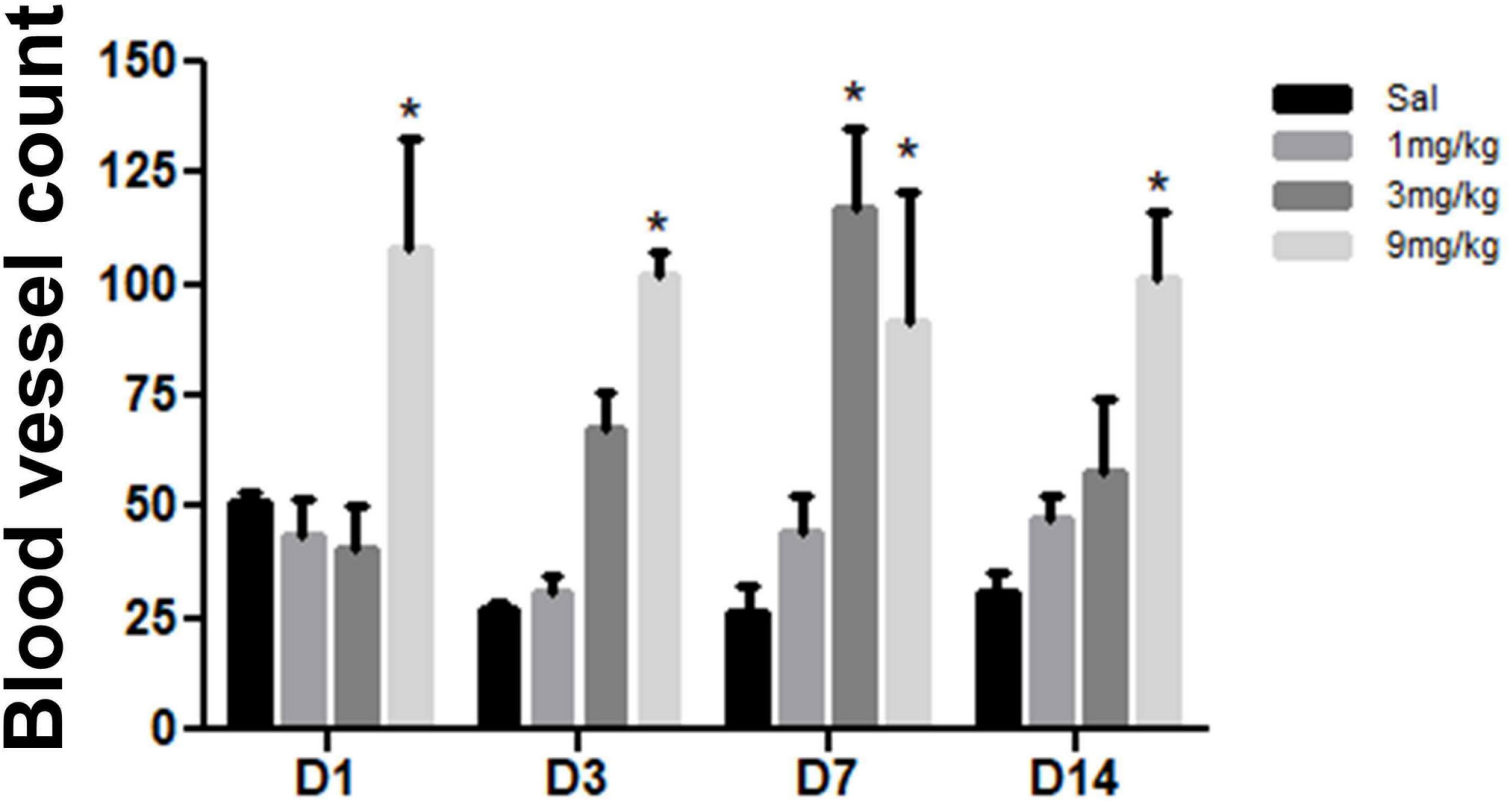
Immunoexpression of CD31 in ulcerated buccal mucosa of Wistar rats treated with alogliptin. *Source:* Data from the study. *p < 0.05 versus saline; (mean ± SEM; Two‐way ANOVA/Bonferroni test).

CD31 expression: Peak expression occurred on Day 1 in the control and GTA9 groups, Day 14 in GTA1, and Day 7 in GTA3. The GTA9 group showed significantly higher CD31 immunostaining on Days 1 (107.8 ± 25.0 cells/mm^2^), 3 (102.0 ± 5.7 cells/mm^2^), 7 (91.6 ± 29.2 cells/mm^2^), and 14 (101.7 ± 14.6 cells/mm^2^) compared to the saline group (51.0 ± 2.7, 26.8 ± 2.0, 26.3 ± 5.8, and 31.2 ± 4.1 cells/mm^2^, respectively). Additionally, the GTA3 group exhibited significantly higher CD31 immunostaining on Day 7 (117.3 ± 18.1 cells/mm^2^) compared to the control group (26.3 ± 5.8 cells/mm^2^) (*p* < 0.001) (Figure 6 and Table S1).

## 4 Discussion

The innate immune system plays a critical role in wound healing by recognizing and eliminating pathogenic microorganisms, preventing colonization and subsequent infections that hinder recovery [5, 27]. In the present study, from Day 1 post‐ulceration, the high‐dose alogliptin group (9 mg/kg/day) had significantly higher microbial counts on the ulcer surface. This persisted until Day 3 when the GTA3 group also showed elevated CFU/mL compared to the untreated group. This suggests impaired bacterial recognition in these groups, allowing ulcer colonization. El‐Sahar et al. [16] observed that alogliptin appears to interfere with mechanisms associated with TLR4 activation, which may compromise bacterial recognition mediated by this receptor. In this study, the results show that alogliptin administration is associated with downregulation of TLR4, as well as TLR2 in the group treated with the highest dose (9 mg/kg/day).

Ulcers in the high‐dose alogliptin group (9 mg/kg/day) were significantly larger from Day 3, persisting until Day 7. The GTA3 group also exhibited increased ulcer areas compared to controls at this time point. These findings are reinforced by microscopic analysis, where all alogliptin‐treated groups had significantly higher histopathological scores on Days 7 and 14 compared to the control group (except GTA1 on Day 7). Immunohistochemical analysis showed that groups receiving higher alogliptin doses (3 and 9 mg/kg/day) exhibited significantly reduced TGF‐β immunostaining, culminating in diminished collagen deposition by the final protocol day in the 9‐mg/kg/day group. These results indicate delayed healing, likely due to compromised innate immune responses to microorganisms colonizing the ulcer surface. The 3‐mg/kg/day dose corresponds to the human equivalent, highlighting the risk of delayed healing in patients using alogliptin or other drugs in this class.

Resident or tissue macrophages are the primary innate immune cells responding to pathogenic bacteria. They are activated through interaction with bacterial LPS via TLR4 on their surface. Upon microbial challenge, activated macrophages release cytokines and mediators critical for recruiting phagocytic cells, such as IL‐8, a chemotactic factor for neutrophils, initiating the fight against pathogens [28, 29]. However, it has already been demonstrated in vitro [12] that alogliptin inhibits pro‐inflammatory cytokine expression in mononuclear cells (macrophages). Similar results were observed in studies with sitagliptin and anagliptin, other DPP‐4 inhibitors, which suppressed TLR4 expression in macrophages [30, 31]. Inhibition of resident macrophages impairs cytokine production and neutrophil chemotaxis [32].

In this study, TLR4 immunostaining was reduced in groups receiving higher alogliptin doses (3 and 9 mg/kg/day) by Day 3, as was TLR2 in the 9‐mg/kg/day group. This led to decreased neutrophil chemotaxis over the experimental protocol, becoming significant on Day 7, when the high‐dose group (9 mg/kg/day) had markedly fewer polymorphonuclear cells compared to the saline group. A study using a DPP4 inhibitor showed that the drug interferes with neutrophil migration [33]. Reduced TLR4 expression may be linked to diminished release of pro‐inflammatory cytokines by tissue macrophages, hindering neutrophil recruitment to the ulcer site.

Neutrophils are critical in combating bacterial infections and are the first cells recruited after tissue injury. Initially recruited by damage‐associated molecular patterns (DAMPs), their chemotaxis is amplified in response to pathogen‐associated molecular patterns (PAMPs). Their primary role is wound debridement, removing dead cells and phagocytosing bacteria [34]. Neutropenia increases infection susceptibility [35] underscoring these cells' importance in fighting pathogens.

Neutrophils' role in infection control and wound healing remains under study, particularly NETosis, a form of neutrophil cell death forming neutrophil extracellular traps (NETs). In this mechanism, neutrophils release chromatin and antimicrobial proteins from their granules, exposing microorganisms to these proteins for elimination. Studies show that impaired NET formation increases bacterial dissemination [35, 36].

Given neutrophils' primary role, their failure to control bacterial colonization recruits other phagocytes, such as monocytes, which become inflammatory macrophages to curb infection [37]. Normally, these macrophages migrate to the wound after neutrophils, recruited by PAMPs and DAMPs, to phagocytize microorganisms and apoptotic neutrophils [38, 39]. By Day 3, mononuclear cell counts were higher in the high‐dose alogliptin groups (3 and 9 mg/kg/day) compared to controls. This likely resulted from reduced tissue macrophage activity and impaired neutrophil migration, prompting the recruitment of inflammatory macrophages and lymphocytes by resident cells (mast cells, dendritic cells, and stromal cells) to compensate for initial phagocytosis deficits [33]. However, these macrophages' activation also depends on LPS recognition via TLR4 [40]. Alogliptin‐induced TLR downregulation likely hindered the activation of migrated macrophages and lymphocytes, contributing to exacerbated bacterial colonization.

Persistent neutrophil deficits and microbial colonization likely contributed to ulcer chronicity. Excessive bacterial burden disrupts normal healing by delaying the proliferation and remodeling phases, which are essential for fibroblast activity and collagen synthesis [41, 42, 43].

Activated macrophages are key to fibroblast migration and pro‐fibrotic factor expression in wounds [44, 45]. However, growth factor expression requires macrophage activation and transition to an anti‐inflammatory, pro‐repair M2 phenotype. M2 macrophages facilitate healing by releasing TGF‐β, which promotes fibroblast migration [41] and stimulates proline synthesis for collagen production [45]. TLR4 downregulation affects macrophage activation and TGF‐β release [46] demonstrating fewer TGF‐β‐expressing macrophages in TLR4‐deficient mice with skin wounds. Similarly, TLR4 inhibition downregulated TGF‐β in hypertrophic scar models [47]. Kabel, Arab, and Elmaaboud [48] reported reduced TGF‐β levels in alogliptin‐treated rats, aligning with our findings of sustained TGF‐β reduction in high‐dose groups (9 mg/kg/day from Day 3; GTA3 from Day 7). TGF‐β is pivotal for collagen synthesis, and its deficiency directly impedes collagenesis [41]. As observed, the 9‐mg/kg/day group had less collagen deposition than controls by the protocol's end. Thus, the hypoexpression of TLR2/TLR4 and TGF‐β, associated with collagen reduction, was linked to delayed wound healing.

The 9‐mg/kg/day group showed elevated CD31 immunostaining from Day 1 onward. Despite the increased vascular density, oral mucosal healing remained delayed in experimental groups. Alogliptin and other DPP‐4 inhibitors enhance cardiac vascularization, though mechanisms remain unclear [49, 50]. Additionally, activated macrophages regulate angiogenesis by secreting anti‐angiogenic factors like endostatin, preventing excessive vascularization [51, 52]. Alogliptin may have increased CD31 expression by impairing macrophage regulatory activity.

Alogliptin monotherapy does not induce hypoglycemia or significant weight changes [53]. However, on Day 14, high‐dose groups (3 and 9 mg/kg/day) had reduced weight gain compared to saline‐treated controls. Oral ulcers are highly painful, potentially causing dysphagia and impairing food intake [54, 55]. This study suggests that delayed healing in high‐dose groups led to larger buccal mucosal lesions, likely reducing food intake and weight. Higher discomfort scores in these groups may reflect intraoral pain.

Finally, impaired ulcer healing was linked to bacterial colonization on the wound surface. While humans' oral microbiota is dominated mainly by *Streptococci* [9, 56], rodents harbor *Streptococci*, *Staphylococci*, and other Gram‐positive/‐negative cocci [57]. CFU counts confirmed the bacterial presence in buccal mucosa ulcers of all animals; however, the lack of microbial profiling through selective/anaerobic media or profiling (16S/qPCR) is a study limitation. Future work should include microbial identification and observational studies. Other limitations of the study include no use of antibiotics/topical antiseptic/TLR agonist, no measurement of DPP‐4/GLP‐1 activity, and increased CD31 without functional angiogenesis assays.

In conclusion, alogliptin may be related to delayed healing of oral ulcers by maintaining inflammation, reducing TGF‐β expression, and impairing collagen deposition. These effects may be driven by increased microbial density resulting from TLR4/TLR2 downregulation.

## Author Contributions

**Maria Imaculada de Queiroz Rodrigues:** conceptualization, methodology, investigation, visualization, writing – original draft. **Joyce Ohana de Lima Martins:** investigation and visualization. **Debora de Souza Collares Maia Castelo‐Branco:** methodology, validation, supervision. **Paulo Goberlânio de Barros Silva:** conceptualization, methodology, formal analysis, supervision, validation, writing – review and editing. **Fabrício Bitu Sousa:** validation, supervision, methodology. **Mário Rogério Lima Mota:** validation, supervision, methodology. **Ana Paula Negreiros Nunes Alves:** conceptualization, methodology, project administration, supervision, writing – review and editing.

## Funding

The authors have nothing to report.

## Ethics Statement

This work followed the ARRIVE checklist and was accepted by the Animal Use Ethics Committee (CEUA) of the Federal University of Ceará (Protocol No. 9982290120).

## Conflicts of Interest

The authors declare no conflicts of interest.

## Supporting information


**Table S1:** Immunoexpression of TL2, TLR4, TGF‐β, and CD31 in ulcerated buccal mucosa of Wistar rats treated with alogliptin.

## Data Availability

The data will be made available upon request to the corresponding author.
