# Supplementary material for: Alogliptin Delays the Healing of Traumatic Oral Ulcers in the Buccal Mucosa of Wistar Rats
Source: Fundam Clin Pharmacol. 2026 Apr 23;40:e70090. doi: 10.1111/fcp.70090 (PMC13105823; doi:10.1111/fcp.70090)
Supplement: Supplementary file 1 — Table S1: Immunoexpression of TL2, TLR4, TGF‐β, and CD31 in ulcerated buccal mucosa of Wistar rats treated with alogliptin. [file FCP-40-0-s001.docx]

Supplementary table – Immunoexpression of TL2, TLR4, TGF-β and CD31 in ulcerated buccal mucosa of Wistar rats treated with Alogliptin.

|  |  | **Alogliptin dosage** | | |  |
| --- | --- | --- | --- | --- | --- |
|  | Saline | 1 mg/Kg | 3 mg/Kg | 9 mg/Kg | **p-Value** |
| **TLR2** |  |  |  |  |  |
| 1D | 926.5±51.0 | 847.8±62.1 | 784.5±95.9 | **525.4±82.0*** | ***0.001^a^*** |
| 3D | 808.5±73.0 | 763.3±60.6 | 579.5±132.1 | **485.0±145.6*** |  |
| 7D | 581.4±50.5 | 544.2±63.3 | 597.7±103.2 | 395.2±109.2 |  |
| 14D | 379.0±127.3 | 345.0±68.3 | 206.6±55.9 | 235.2±46.4 |  |
| **TLR4** |  |  |  |  |  |
| 1D | 885.3±82.8 | 733.7±57.1 | 727.5±71.1 | 717.8±80.5 | ***0.001***^a^ |
| 3D | 697.0±115.2 | 740.3±14.5 | **332.0±114.2*** | **404.7±129.4*** |  |
| 7D | 220.6±33.6 | 82.8±33.0 | 164.5±27.3 | 56.3±14.8 |  |
| 14D | 104.2±39.6 | 110.5±42.8 | 62.6±26.5 | 60.6±16.5 |  |
| **TGF-β** |  |  |  |  |  |
| 1D | 438.0±32.9 | 447.6±63.0 | 426.2±35.9 | 300.0±82.0 | ***<0,001^a^*** |
| 3D | 397.3±30.0 | 420.5±54.0 | 281.8±23.3 | **121.2±25.3*** |  |
| 7D | 362.4±39.0 | 363.0±80.8 | **173.5±16.1*** | **175.5±43.6*** |  |
| 14D | 395.7±26.5 | 340.3±96.3 | **145.0±33.4*** | **155.0±14.9*** |  |
| **CD31** |  |  |  |  |  |
| 1D | 51.0±2.7 | 43.5±8.1 | 40.5±9.7 | **107.8±25.0*** | ***<0,001^a^*** |
| 3D | 26.8±2.0 | 30.5±3.9 | 67.8±8.1 | **102.0±5.7*** |  |
| 7D | 26.3±5.8 | 44.0±9.0 | **117.3±18.1*** | **91.6±29.2*** |  |
| 14D | 31.2±4.1 | 47.3±5.2 | 57.8±16.6 | **101.7±14.6*** |  |

^a^Two-way ANOVA /Bonferroni test.

^*^ p<0,05 versus Saline

Source: Research data from the study.
